# The promotion of the transformation of quiescent gastric cancer stem cells by IL-17 and the underlying mechanisms

**DOI:** 10.1038/onc.2016.291

**Published:** 2016-08-15

**Authors:** Y-X Jiang, S-W Yang, P-A Li, X Luo, Z-Y Li, Y-X Hao, P-W Yu

**Affiliations:** 1 Department of General Surgery, Center of Minimal Invasive Gastrointestinal Surgery, Southwest Hospital, Third Military Medical University, Chongqing, China

## Abstract

Postoperative recurrence and metastasis have crucial roles in the poor prognosis of gastric cancer patients. Previous studies have indicated that gastric cancer originates from cancer stem cells (CSCs), and some investigators have found that a particular subset of CSCs possesses higher metastatic capacity. However, the specific mechanism remains uncertain. In the present study, we aimed to explore the biological functions of the inflammatory cytokine interleukin-17 (IL-17) in gastric cancer metastasis and the distinct IL-17-induced transformation of quiescent gastric CSCs. Our results showed that invasive gastric CSCs were CD26+ and CXCR4+ and were closely associated with increased metastatic ability. The quiescent gastric CSCs, which were CD26− and CXCR4−, were exposed to appropriate concentrations of IL-17; this resulted in the decreased expression of E-cadherin and the increased expression of vimentin and N-cadherin. In addition, the upregulation of IL-17 both *in vitro* and *in vivo* resulted in a significant induction of invasion, migration and tumor formation ability in gastric CSCs compared with the control group, which was not treated with IL-17. Further experiments indicated that the activation of the downstream phosphorylated signal transducer and activator of transcription 3 (STAT3) transcription factor pathway was facilitated by IL-17. On the contrary, the downregulation of STAT3 by the specific inhibitor Stattic significantly reversed the IL-17-induced epithelial–mesenchymal transition (EMT)-associated properties of quiescent gastric CSCs. Moreover, tumorigenesis and metastasis were suppressed. Taken together, we suggest that IL-17 is positively correlated with the transformation of quiescent gastric CSCs into invasive gastric CSCs and that targeting IL-17 may emerge as a possible novel therapeutic strategy for gastric cancer.

## Introduction

Gastric cancer is one of the most common gastrointestinal malignancies, and the morbidity and mortality of patients with gastric cancer remain high. Key biological characteristics of gastric cancer include invasion and metastasis, which are the main factors that are responsible for postoperative recurrence and the development of therapeutic resistance. Studies focused on the mechanisms that underlie the invasion and metastasis of gastric cancer have attracted extensive attention, but these mechanisms have not been fully elucidated.^1, 2^

An increasing amount of evidence supports the cancer stem cell (CSC) theory. This theory proposes that CSCs serve not only as the basis for the development and progression of tumors but also as the primary reason for tumor recurrence and metastasis. Recent studies have demonstrated that CSCs are heterogeneous. CSCs are divided into different subsets including quiescent CSCs and invasive CSCs based on their distinct characteristics. Invasive CSC is a major motivation to cancer metastasis. Additionally, quiescent CSCs may differentiate into invasive CSCs in specific microenvironments, thereby promoting distant tumor metastasis.^3, 4^

As a proinflammatory cytokine, IL-17 is primarily secreted by T helper type 17 cells and neutrophils. IL-17 is a homodimer that is composed of 155 amino acids with a relative molecular mass that ranges from 15 to 22 × 10^3^ Da. The IL-17 protein contains an N-terminal signal peptide. A variety of chronic inflammatory diseases, such as inflammatory bowel disease, *Helicobacter pylori* infection, hepatitis B virus infection and cryptogenic chronic prostatitis, are capable of inducing tumors known as inflammation-associated neoplasias. Moreover, increased IL-17 expression has been observed in these tumors.^5, 6, 7, 8^ Our previous study found that IL-17 was highly expressed in gastric cancer tissues and that the IL-17 expression level was closely related to the depth of infiltration, lymph-node metastasis and tumor–node–metastasis staging of gastric cancer. Gastric cancer patients with a high level of IL-17 expression showed a poor prognosis. However, the determination of the specific underlying mechanisms requires further investigation.^9^

In the present study, different subsets of gastric CSCs were successfully isolated. *In vivo* and *in vitro* experiments were then conducted on the isolated gastric CSCs. We found that IL-17 was able to promote epithelial–mesenchymal transition (EMT)-like transformation of gastric CSCs. Further experiments indicated that the signal transducer and activator of transcription 3 (STAT3) protein was a downstream signaling molecule that was involved in IL-17 activity.

## Results

### Successful isolation and identification of gastric CSCs

**Figure 1:**
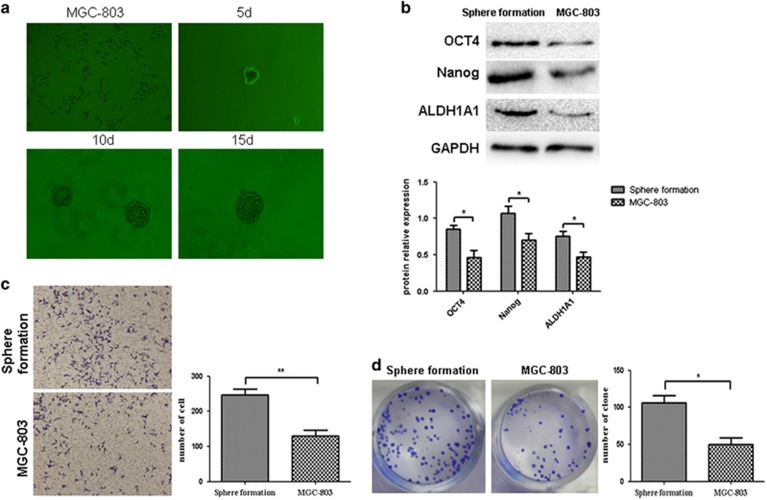
Successful isolation of gastric CSCs and evaluation of the related stemness phenotypes. (**a**) Isolation of gastric cancer cell spheroids with a diameter between 40 and 100 μm using a sphere culture system. (**b**) Verification of stemness by WB. Compared with the ordinary cultured cells, the spheroid-forming cells expressed higher levels of the stemness markers OCT4 (*P<0.05), Nanog (*P<0.05) and ALDHA1 (*P<0.05). (**c**) The Transwell invasion assay demonstrated that the spheroid-forming cells showed greater invasive capability (**P<0.01). (**d**) The plate-based clonogenic assay demonstrated that the spheroid-forming cells possessed a more potent colony-forming ability (*P<0.05).

Spheroid-forming cells were isolated using a sphere culture system and were compared with the differentiated adherent cells (Figure 1a). The results showed that the expression levels of the stemness factors OCT4, Nanog and ALDHA1 were significantly higher in the spheroid-forming cells than in the differentiated MGC-803 cells (*P*<0.05) (Figure 1b). Additionally, the spheroid-forming cells exhibited an increased invasive capability when the invasive capability was compared between the two types of cells using the Transwell invasion assay (Figure 1c). The plate-based clonogenic assay also showed that compared with ordinary cultured cells, the spheroid-forming cells possessed a more potent colony-forming ability (Figure 1d). The above results demonstrated that gastric CSCs could be isolated using the sphere culture system. Phenotypes of the isolated spheroid-forming cells were consistent with those of stem cells.

### Sorting and identification of gastric CSC subsets

**Figure 2:**
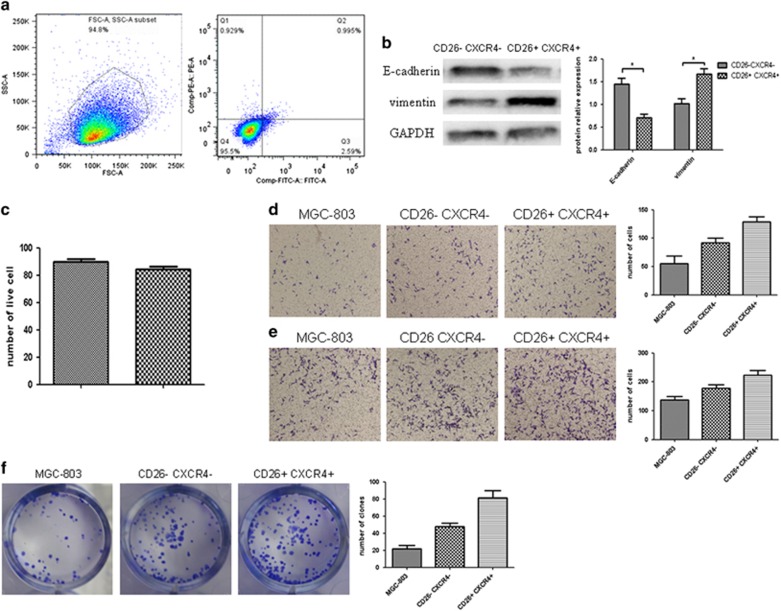
(**a**) The results of flow cytometry-based cell sorting indicated that the CD26+ CXCR4+ double-positive cells (Q2), CXCR4-positive cells (Q1+Q2) and CD26-positive cells (Q4+Q2) accounted for 0.995, 1.924 and 3.585% of the gastric cancer cells, respectively. (**b**) WB results showed that CD26+ CXCR4+ gastric CSCs exhibited a drastic decrease in E-cadherin expression (*P<0.05) and a significant increase in vimentin expression (*P<0.05). (**c**) The trypan blue exclusion assay showed the different cell viability after cell sorting (*P<0.05). (**d**) The Transwell invasion assay showed that the invasive capability of the quiescent gastric CSCs was lower than that of the invasive gastric CSCs. (**e**) The Transwell migration assay showed that the migration ability of the quiescent gastric CSCs was lower than that of the invasive gastric CSCs. (**f**) The plate-based clonogenic assay also demonstrated that the clonal proliferative capacity of the quiescent gastric CSCs was lower than that of the invasive gastric CSCs.

Different stem cell subsets display distinct cell surface markers. Recent studies have determined that the biological functions of CD26 are related to metastatic capacity in colorectal CSCs,^10, 11^ while CXCR4 is regarded as a key receptor that is closely involved in tumor invasion and metastasis. Therefore, we selected CD26 and CXCR4 as markers of invasive stemness^12, 13^ and performed flow cytometry-based cell sorting on the cell spheroids based on the expression of CD26 and CXCR4. The CD26-positive cells and CXCR4-positive cells accounted for 2.59 and 0.929% of the gastric cancer cells, respectively, and the double-positive cells and double-negative cells accounted for 0.995 and 95.5% of the gastric cancer cells, respectively (Figure 2a). In the present study, the CD26+ CXCR4+ double-positive cells were defined as invasive gastric CSCs, whereas the CD26− CXCR4− double-negative cells were defined as quiescent gastric CSCs. To test cell viability, we conducted a trypan blue exclusion assay, and the results showed that cell viability was 95.58±0.87% before cell sorting and 94.96±0.76% after cell sorting (*P*>0.05) (Figure 2c), which indicated that flow cytometry-based cell sorting had virtually no effect on cell viability. The western blotting (WB) results revealed a remarkable increase in vimentin protein expression and a decrease in E-cadherin expression in CD26+ CXCR4+ gastric CSCs, compared with the CD26− CXCR4− group (Figure 2b). We investigated the invasive capabilities and colony-forming abilities of the sorted cell subsets and found that the invasive capability and the clonal proliferative capacity of the quiescent gastric CSCs were significantly lower than those of the invasive gastric CSCs (*P*<0.05) (Figures 2d–f).

### IL-17 promotes the invasive transformation of quiescent gastric CSCs

**Figure 3:**
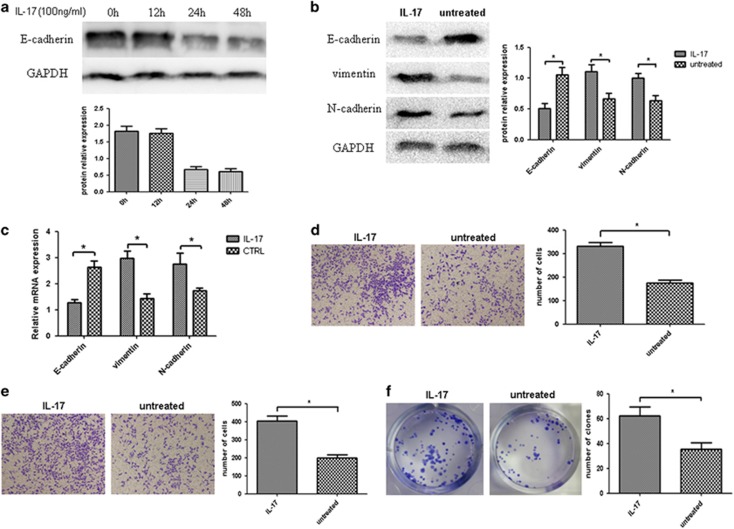
IL-17 promotes the invasive transformation of quiescent gastric CSCs. (**a**) The quiescent gastric CSCs exhibited the most significant decrease in the level of E-cadherin expression after treatment with 100 ng/ml of IL-17 for 24 h. (**b**) IL-17-treated quiescent gastric CSCs exhibited a drastic decrease in E-cadherin expression (*P<0.05) and a significant increase in vimentin (*P<0.05) and N-cadherin expression (*P<0.05). (**c**) The reverse transcription PCR (RT–PCR) results showed that IL-17 treatment enhanced EMT in the quiescent gastric CSCs compared with the control group (*P<0.05). (**d**) The difference in the invasive capability between the treated group and the control group was significant (*P<0.05). (**e**) The migration assay showed that invasive capability was significantly enhanced in the IL-17-treated quiescent gastric CSCs (*P<0.05). (**f**) The difference in the clonal proliferative capacity between the treated group and the control group was significant (*P<0.05).

To explore the ability of IL-17 to promote the transformation of quiescent gastric CSCs, the cells were treated with exogenous IL-17, and changes in the biological characteristics of the cells were examined. We selected the appropriate concentration of IL-17 (100 ng/ml) based on the literature,^14^ then we successfully established the gastric CSC that stably overexpresses IL-17 (Supplementary Figures 1A and B). In the present study, total proteins were extracted from the cells after treatment with IL-17 for 0, 12, 24 and 48 h. Then, WB was performed to examine the changes in E-cadherin protein expression and to screen for the optimal duration of IL-17 treatment (Figure 3a). After the quiescent gastric CSCs were treated with the established concentration of IL-17 for an optimal period of time, markers of invasiveness were analyzed by flow cytometry. Additionally, changes in the expression of EMT-related makers, the invasive capability and the clonal proliferative ability were examined. The results showed that E-cadherin expression was decreased and that N-cadherin and vimentin expression was increased in the quiescent gastric CSCs after IL-17 treatment (Figures 3b and c). The Transwell migration and invasion assays showed that the migratory and invasive capabilities of the quiescent gastric CSCs were enhanced after IL-17 treatment compared with the control group (Figures 3d and e). The clonogenic assay demonstrated that the clonal proliferative capacity of the quiescent gastric CSCs increased after IL-17 treatment and that significant differences existed between the IL-17-treated group and the control group in terms of clonal proliferative capacity (*P*<0.05) (Figure 3f).

### IL-17 enhances tumorigenesis and metastasis of quiescent gastric CSCs *in vivo*

**Figure 4:**
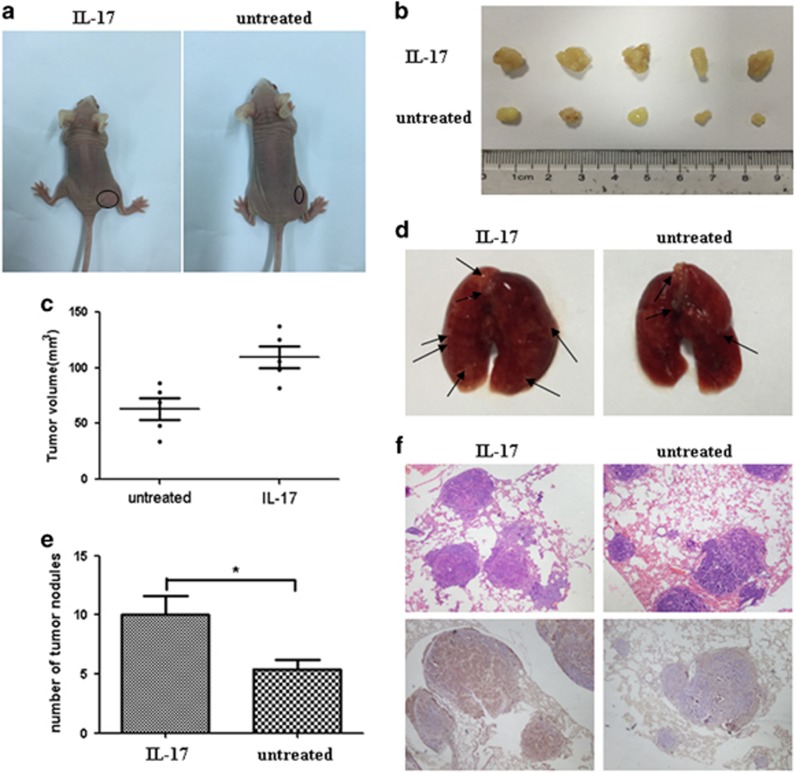
*In vivo* experiments demonstrate that IL-17 promotes the tumorigenesis and invasive transformation of quiescent gastric CSCs. (**a**) Subcutaneously established quiescent gastric CSC-derived tumors in nude mice. (**b**) Images of subcutaneous tumor xenografts in nude mice. (**c**) A scatterplot compares the volumes of tumor xenografts (*P<0.05). (**d**) Gross lung specimens derived from the nude mice were examined. The lung tissues derived from the experimental group contained an increased number of tumor nodules compared with the control group. (**e**) Detailed information about the number of lung metastases. The results showed that the differences were significant (*P<0.05). (**f**) H&E and immunohistochemical staining of the lung specimens demonstrated that the tumor nodules were derived from the tumor cells injected through the tail vein.

To investigate the transforming effect of IL-17 on quiescent gastric CSCs, two *in vivo* experimental models were established to test the tumor xenograft volumes: subcutaneous injection of cancer cells into nude mice for tumorigenic assay and tail vein before the examination for the number of lung metastases. The experiment showed that the quiescent gastric CSCs exhibited increased invasive and metastatic capacities and revealed the tumorigenicity of these cells after treatment with 100 ng/ml of IL-17 for 24 h. As shown in Figure 4, the IL-17-treated quiescent gastric CSCs induced larger tumor xenografts (*P*<0.05) (Figures 4a–c, Supplementary Figures 2A and B). Moreover, the number of lung metastatic foci was significantly increased in mice that were injected with IL-17-treated cells compared with mice that were injected with control cells (*P*<0.05) (Figures 4d–f).

### IL-17 exerts a promoting effect on quiescent gastric CSCs through STAT3

**Figure 5:**
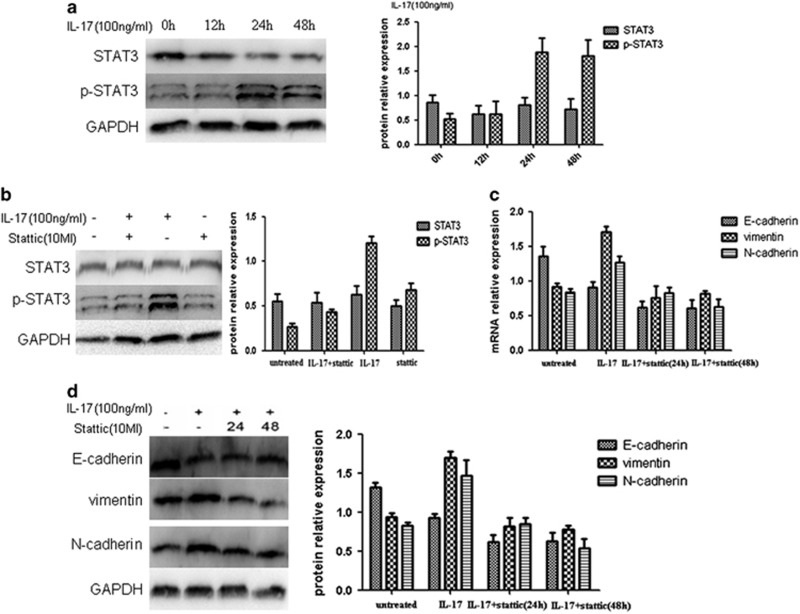
IL-17 exerts a promoting effect on quiescent gastric CSCs through STAT3. (**a**) Relative changes in the protein expression levels of p-STAT3 and STAT3 were examined in cells treated with 100 ng/ml of IL-17 for various lengths of time. (**b**) The relative protein expression levels of p-STAT3 and STAT3 were determined in cells treated with Stattic and were compared with the corresponding expression in the control group. (**c**) Examination of changes in EMT-related molecules after treatment with IL-17 and Stattic by reverse transcription PCR (RT–PCR). (**d**) Examination of changes in EMT-related molecules after treatment with IL-17 and Stattic by WB.

Based on our previous findings and on the relevant literature, we hypothesized that STAT3 might act as a downstream regulator of IL-17 and that STAT3 might have a role in the transformation of quiescent gastric CSCs.^5^ In the present study, STAT3 expression was examined in IL-17-treated quiescent gastric CSCs at different time points (Figure 5a). In addition, changes in the expression of EMT markers were investigated after inhibition of the STAT3 signaling pathway. WB results showed that IL-17 upregulated the expression of p-STAT3. However, no significant difference was observed in the expression of unphosphorylated STAT3. Moreover, the maximum increase in STAT3 expression was achieved after treatment of the quiescent gastric CSCs with 100 ng/ml of IL-17 for 24 h, which was consistent with the result of the previous screening experiment. The addition of the STAT3 signaling pathway inhibitor Stattic resulted in the downregulation of STAT3 expression (Figure 5b). Further experiments demonstrated corresponding changes in EMT markers. Stattic reduced the promoting effect of IL-17 on the transformation of quiescent gastric CSCs. The results demonstrated that IL-17 promoted the EMT-related transformation of quiescent gastric CSCs through STAT3 (Figures 5c and d).

### IL-17 increases the metastatic and clonogenic capability of quiescent gastric CSCs via activation of the STAT3 pathway

**Figure 6:**
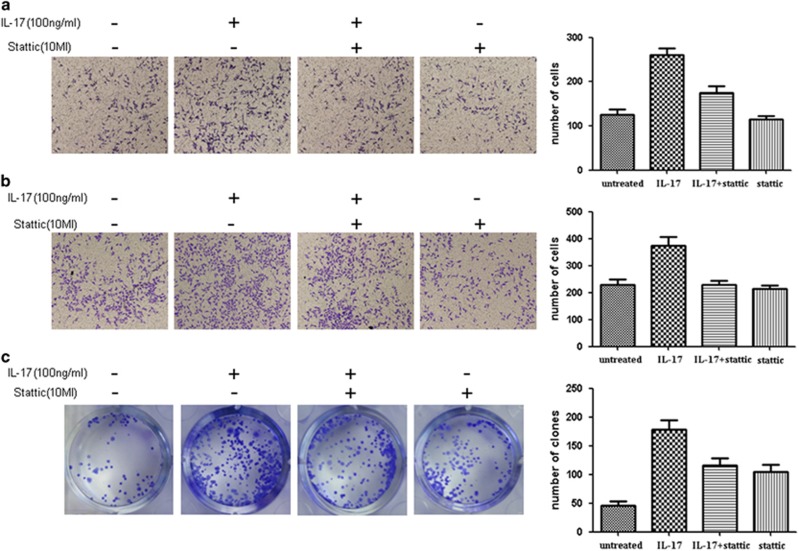
(**a**) Examination of the changes in the invasiveness of the cells using a Transwell assay. (**b**) Examination of the changes in the migration ability of the cells using a Transwell assay. (**c**) Examination of the changes in the clonal proliferative capacity of the cells using a plate-based clonogenic assay.

To further explore the effect of IL-17 and STAT3 on the metastatic and clonogenic capabilities of quiescent gastric CSCs, Transwell invasion and migration assays as well as a cell clonogenic assay were conducted in the four groups (control, IL-17, IL-17+ Stattic and Stattic). The results showed that as quiescent gastric CSCs were treated with Stattic, the IL-17-induced invasiveness and increased migration ability were significantly reversed (*P*<0.05) (Figures 6a and b). Additionally, decreased colony formation was observed in the IL-17+ Stattic group compared with the IL-17 group (*P*<0.05). The suppression of IL-17 indicated that STAT3 might be extremely crucial in the metastatic and clonogenic capabilities of quiescent gastric CSCs (Figure 6c).

## Discussion

Stem cells are considered key players in the processes of tumor invasion and metastasis, and recent studies have shown that CSCs display heterogeneity.^15, 16, 17^ The heterogeneity of CSCs is primarily reflected in the three aspects discussed below. First, different subsets of stem cells express distinct surface markers. Wright *et al.*^18^ found that breast CSCs could be divided into CD44+/CD24− and CD133+ subsets based on differences in surface marker expression. Both subsets possess tumor stem cell-like characteristics that do not overlap. Second, the heterogeneity of CSCs is reflected in the differences in the cell characteristics. Specifically, some stem cell subsets possess a strong invasive capability, whereas other stem cell subsets are in a quiescent state and do not differentiate.^19^ Mani *et al.*^20^ conducted a study on human colon cancer liver metastases and found that a portion of the cancer cells displayed stem cell-like morphologies and expressed stem cell markers. However, the cells displayed extremely weak tumorigenicity and a poor invasive capability and only played a role in the maintenance of primary tumor growth. Therefore, these stem cells were defined as quiescent stem cells.^21^ Third, the quiescent state of stem cells is not permanent. Under appropriate external environmental stimuli, quiescent stem cells may undergo invasive transformation and become invasive stem cells.^22^ Therefore, an investigation of the factors that promote quiescent stem cell transformation is of great clinical significance.

Using previously established methods for the isolation and identification of glioma stem cells,^23^ we isolated (enriched) gastric CSCs from the gastric cancer cell line MGC-803 using serum-free conditioned medium and a sphere culture system. After optimization of a variety of conditions, gastric CSCs were successfully obtained and identified. The isolated and enriched gastric CSCs exhibited clear ‘stemness' characteristics. CXCR4 and CD26 have been recognized as gastrointestinal CSC markers by experts in the CSC field.^24, 25^ We found that CD26+ cells accounted for ~3.6% of the isolated gastric CSCs and that CXCR4+ cells accounted for ~1.9%. The remainder of the isolated gastric CSCs expressed no or low levels of CXCR4 and CD26. According to the relevant literature, we defined the CD26+ CXCR4+ double-positive cells as invasive gastric CSCs and the CD26− CXCR4− double-negative cells as quiescent gastric CSCs.^26, 27^ We performed an invasion assay to examine the invasive capabilities of the two gastric CSC subsets and found that the double-positive gastric CSCs possessed a significantly increased invasive capability and clonal expansion capacity compared with the quiescent gastric CSCs. The results indicated that gastric CSCs were not homogeneous and consisted of both quiescent gastric CSCs and invasive gastric CSCs, which was consistent with the results of a number of cancer studies.^28, 29^ Thus, we suggested that CXCR4 and CD26 may be appropriate markers that may be used to sort the gastric CSC subsets. Our experimental results also showed that E-cadherin expression was significantly decreased and that vimentin expression was increased in the invasive gastric CSC subset compared with the quiescent gastric CSC subset, which indicates that the invasive gastric CSCs acquired EMT-like characteristics. Numerous studies have found that tumor cells may exhibit stem cell characteristics when they undergo EMT in an inflammatory microenvironment. Certain CSCs may acquire EMT-like characteristics under the induction of inflammatory cytokines and display high invasive and metastatic capacities. However, the specific underlying mechanisms remain unclear.

The stimulating effect of inflammatory microenvironments, inflammatory cells and inflammatory cytokines on EMT of tumors has been recognized. Recently, accumulating evidence has demonstrated that tumor cells acquire stem cell characteristics during EMT such as redifferentiation into epithelial tumor cells and the ability to self-renew.^30, 31^ Sansteban *et al.*^32^ demonstrated that CD8+ T cells were able to induce an EMT-like transformation of tumor cells. The transformed tumor cells exhibited stem cell characteristics, including tumorigenic potential, the ability to regenerate epithelial tumors and enhanced resistance to radiochemotherapy. In the present study, quiescent gastric CSCs were treated with an appropriate concentration of exogenous IL-17 for 24 h. Significant differences were observed in the invasive capability, migration capability and expression of EMT-related markers between the IL-17-treated quiescent gastric CSCs and the untreated quiescent gastric CSCs. Moreover, an elevated tumorigenic capacity was also identified by a cell clonogenic assay. We also revealed that IL-17 could prompt the formation of neoplasms in the form of subcutaneous xenografts and lung metastatic foci in a mouse model. These results indicated that IL-17 may promote the transformation of quiescent gastric CSCs into invasive gastric CSCs *in vivo* and *in vitro*.

The interleukin family is involved in a large number of regulatory networks that operate in tumor cells. For example, interleukin 6 (IL-6)/IL-6 receptor (IL-6R)-mediated signaling pathways are involved in the regulation of EMT in glioma cells. After it binds to the corresponding cell membrane receptor, the IL-6 molecule first activates the Janus tyrosine kinase/STAT6 cascade. Activated Janus tyrosine kinase phosphorylates a tyrosine residue (705th amino acid) in the STAT3 molecule and regulates the expression of related downstream genes.^33, 34^ STAT3 is an important member of the STAT family. STAT family members are regarded as a class of bifunctional molecules that are involved not only in signal transduction but also in the activation of gene expression. Currently, STAT3 signaling is believed to be one of the signal transduction pathways that is most closely related to the occurrence of malignant tumors. Excessive activation of STAT3 promotes tumor EMT. Additionally, the transcriptional activity of STAT3 is a prerequisite for the regulation of tumor invasion and metastasis.^35, 36, 37^ Studies on the relationship between IL-17 and interferon gamma (IFN-γ) have found that the expression level of IL-17 is positively correlated with the degree of activation of the STAT3 signaling transduction pathway.^38^ As some similar structural domains exist in both of IL-17 and IL-6, we deduced that the mechanism underlying IL-17-mediated STAT3 activation may be similar to the mechanism underlying IL-6-induced STAT3 activation. The present study found that the STAT3 pathway was activated after treatment of the gastric CSCs with exogenous IL-17. Administration of a STAT3 inhibitor reduced EMT in gastric CSCs and decreased the invasiveness, migration capability and proliferative capacity of gastric CSCs. The results demonstrated that IL-17 promotes an EMT-like transformation of gastric CSCs through the STAT3 pathway.

The present study revealed for the first time the distinct cell subsets among gastric CSCs. The cellular characteristics of the different subsets were preliminarily explored. Additionally, the specific role of IL-17 in the EMT-like transformation of gastric CSCs was investigated on the basis of our previous study. Understanding the characteristics of the subset of cells that can be transformed by IL-17 or the mechanisms that underlie the inhibition of the transformation of other quiescent gastric CSCs will be the direction of our future research and cancer treatment plans.

## Materials and methods

### Cell culture and formation of tumor spheres

The human gastric cancer cell line MGC-803 was purchased from the American Type Culture Collection (ATCC). After thawing, the cells were cultured in Dulbecco's Modified Eagle's Medium (DMEM) supplemented with 10% fetal bovine serum, 100 U/ml penicillin and 100 U/ml streptomycin at 37 °C in a 5% CO_2_ incubator. In all, 2 × 10^4^ cells in the logarithmic growth phase were seeded into 100-mm ultra-low attachment dishes and were cultured in conditioned DMEM/F12 stem cell medium (1:1, Gibco, Grand Island, NY, USA) supplemented with epidermal growth factor (Sigma, St Louis, MO, USA), basic fibroblast growth factor (Sigma) and B27 supplement (1 × , Invitrogen, Carlsbad, CA, USA). Tumor sphere dissociation and collection were conducted as previously described.^39, 40^ Healthy, exponentially growing cells were used in the subsequent experiments.

### Reverse transcription PCR

RNA was extracted from the tumor spheres using TRIzol reagent in accordance with the manufacturer's instructions and was quantified using ultraviolet spectrophotometry. Complementary DNA was synthesized using the PrimeScript RT Reagent Kit (Takara Biomedicals, Kusatsu, Japan) according to the manufacturer's instructions. All the primer sequences were listed in Table 1. The reaction conditions were as follows: 42 °C for 60 min (1 cycle) followed by 85 °C for 5 min and 4 °C for 5 min (38 cycles). PCR was conducted using SYBR Premix Ex Taq (Takara Biomedicals) according to the manufacturer's instructions. The PCR conditions were as follows: 95 °C for 30 s followed by 48 °C for 30 s and 60 °C for 60 s (40 cycles). Glyceraldehyde-3-phosphate dehydrogenase (GAPDH) was used as the reference gene. The relative gene expression level was determined using the comparative Ct method.

### Cell invasion and migration assay

Transwell culture inserts (8 μm pore size), 24-well plates, Matrigel (Millipore Corporation, Darmstadt, Germany) and pipette tips were pre-cooled at 4 °C. Matrigel was diluted 1:2 in serum-free DMEM. An appropriate amount of Matrigel was added to the upper chamber of the Transwell plates for the invasion assay, while the plates without Matrigel in the upper chamber were used for the migration assay. Then, the chambers were incubated at 37 °C in a 5% CO_2_ incubator for 4–8 h. The treated cells were resuspended into a single-cell suspension and seeded into the upper chamber at a density of 2 × 10^5^ cells/well. Subsequently, 200 ml of serum-free DMEM was added to the upper chamber, and 750 ml of complete DMEM containing 10% fetal bovine serum was added to the lower chamber. The cells were incubated at 37 °C for 24 h in a 5% CO_2_ incubator. The inserts were collected, and the cells were fixed in 4% polyformaldehyde at room temperature (RT) for 20 min and then stained with crystal violet for 30 min. The non-invading cells that remained in the Matrigel layer or on the upper surface of the Transwell insert membranes were removed with a cotton swab. To observe the cells that had invaded through the semipermeable Transwell insert membrane and that had attached to the lower surface of the membranes, the inserts were placed upside-down under an inverted phase contrast microscope. Subsequently, three microscopic fields of view were randomly selected. All of the above experiments were repeated at least three times, and the average number of cells per high-power field was calculated and statistically analyzed.

### Western blotting

The treated cells were washed twice with pre-cooled phosphate-buffered saline (PBS) and then immersed in ice-cold lysis buffer containing 150 mmol/l NaCl, 50 mmol/l Tris-HCl (pH 7.6), 0.1% sodium dodecyl sulfate (SDS), 1% NP-40 and protease inhibitor cocktail. After repeated pipetting, the cells were sonicated for 15–20 min and then centrifuged at 16 000 r.p.m. for 15 min. Total protein lysates were collected and quantified using the Bradford method. The protein samples (50 μg each) were subjected to SDS–polyacrylamide gel electrophoresis (PAGE) and transferred onto nitrocellulose membranes. After blocking with 5% skim milk for 1–2 h, the membranes were incubated with the following primary antibodies (anti-STAT3 antibody (Abcam, Cambridge, MA, USA), 1:4000 dilution; anti-E-cadherin antibody (CST, Beverly, MA, USA), 1:1000 dilution; anti-N-cadherin antibody (CST), 1:1000 dilution; anti-vimentin (CST) antibody, 1:1000 dilution; anti-octamer binding transcription factor 4 (Oct4) antibody (Abcam), 1:1000 dilution; anti-aldehyde dehydrogenase 1 family member A1 (ALDH1A1) antibody (Abcam), 1:1000 dilution; anti-Nanog antibody (Abcam), 1:1000 dilution; and anti-GAPDH antibody (Proteintech, Chicago, IL, USA), 1:5000 dilution. Membranes were incubated at 4 °C overnight and then at RT for 30–60 min. Subsequently, the membranes were washed three times (10 min each) in Tris-buffered saline-Tween-20 (TBST) and were incubated with the corresponding secondary antibodies at RT for 1 h. The protein bands were visualized using an enhanced chemiluminescence (ECL) detection system. All antibodies were purchased from the indicated companies. Moreover, all of the above experiments were repeated at least three times, and the results were statistically analyzed.

### Determination of cell proliferative capacity by clonogenic assay

All groups of cells were treated, counted and transferred to 24-well cell culture cluster dishes (1000 cells per dish). The cells were routinely cultured for 2 weeks before the assessment of colony formation using an inverted microscope. After the removal of the culture medium, the cells were fixed in 4% polyformaldehyde for 15 min and stained with crystal violet for 20 min. The dye was washed away with PBS, and the colonies were observed and counted under an inverted microscope. Cell clusters that contained >50 cells were counted as colonies. Colony formation rate (%)=(number of clones/1000) × 100%.

### *In vivo* experiments

Twenty 4- to 6-week-old nude mice (males) were divided into the following two groups: the quiescent gastric CSC group and the IL-17-treated group. Each group contained five mice. After treatment, the cells were centrifuged (1000 r.p.m. for 5 min) and resuspended in PBS. The establishment of a subcutaneous xenograft tumor model: to construct the subcutaneous tumor model, 0.2 ml of IL-17-treated (100 ng/ml)quiescent gastric CSCs (which were suspended in a 1:1 mixture of PBS and Matrigel at a density of 1 × 10^6^ cells/ml) was injected subcutaneously into the right back of each nude mouse in the experimental group. Mice in the control group were injected with equal amounts of untreated stem cells. Four weeks after the establishment of the model, all mice were killed. The long diameters (*a*) and transverse (*b*) diameters of the tumors were measured using a vernier caliper. Tumor volume was calculated using the following equation: *V*=1/2*ab*^2^ (mm^3^). Establishment of a metastatic tumor model by tail vein injection: the cell density was adjusted to 5 × 10^6^ cells/ml. Each nude mouse was given an injection of 0.2 ml of IL-17-treated (100 ng/ml) quiescent gastric CSCs into the tail vein. For the control group, each nude mouse was injected with 0.2 ml of untreated cell suspension via the tail vein. The nude mice were monitored daily for changes in body weight and other physiological indicators. All the mice were maintained for 6 weeks and then killed by cervical dislocation. The lungs and other organs collected from the mice were examined to determine whether metastatic foci were present. The metastatic foci were counted and confirmed as human gastric cancer xenografts by hematoxylin and eosin (H&E) staining and immunohistochemistry. The data were then statistically analyzed. All mice were given adequate humanitarian care in accordance with NIH animal protection organization guidelines.

### Sorting of stem cell subpopulations by flow cytometry

Cultured stem cell spheroids were dispersed by gentle pipetting. After centrifugation and collection, the cells were washed twice with PBS and resuspended in pre-cooled (4 °C) PBS containing 2% bovine serum albumin. The cell suspension (100 μl each) was mixed thoroughly with fluorescein isothiocyanate-conjugated cluster of differentiation 26 (CD26) (BD Biosciences, Franklin Lakes, NJ, USA) or PE-conjugated C-X-C chemokine receptor type 4 (CXCR4) (BD Biosciences) and incubated at 4 °C in the dark for 30 min. The cells were washed twice with PBS and centrifuged at 2500 r.p.m. for 5 min. After the removal of the supernatant, cell pellets were resuspended in 500 μl of PBS and then examined and sorted using a Becton-Dickinson FaCSaria II system (BD Biosciences, San Jose, CA, USA). The results were statistically analyzed, and the sorted cells were used in the subsequent experiments.

### Cell viability assay

The cells obtained after flow cytometry-based cell sorting were seeded into 24-well culture plates and cultured at 37 °C/5% CO_2_ for 12 h in Earle's Balanced Salt Solution. Then, the cells were digested with an enzyme solution, centrifuged and collected. The cell suspensions (100 μl) were mixed thoroughly with 100 μl of a 0.8% trypan blue solution. At least 200 cells were counted in each sample, and each sample was counted three times. Cells stained blue were counted as dead cells. The dead and live cells were counted rapidly by a cell counter, and the results were statistically analyzed.

### H&E staining and immunohistochemistry

Lung tissues from the nude mice were fixed in 4% formaldehyde for 24 h, embedded in paraffin, and serially sectioned (at a thickness of 5–6 μm). The tissue sections were deparaffinized by washing twice in xylene (10 min each wash) and were rehydrated through a decreasing ethanol gradient (10 min per step). Subsequently, the tissue sections were washed with tap water for 5 min, incubated with 3% H_2_O_2_ at RT for 30 min, washed again with tap water for 5 min and immersed in PBS (0.01 mol/l, pH 7.4) for 5 min. The tissue sections were subjected to antigen retrieval as follows: briefly, the tissue sections were boiled in citrate buffer (0.01 mol/l, pH 6.0) at a temperature above 96 °C for 15 min; the sections were slowly cooled to RT, washed with tap water for 5 min and immersed in PBS for 5 min. After antigen retrieval, the primary antibody solutions were placed onto the sections, which were incubated at RT for 30 min and then at 4 °C overnight. The sections were washed three times with PBS for 5 min each time, incubated with Polymer Helper (included in the PV 9000 Kit Invitrogen) at RT for 60 min and washed three more times with PBS (5 min each wash). Subsequently, the sections were incubated with horseradish peroxidase-conjugated anti-mouse/rabbit IgG (added drop-wise) at RT for 20–30 min, washed three times with PBS for 5 min each time and stained with 3,3′-diaminobenzidine (DAB; preparation: 1 ml of distilled water+1 drop of reagent B+1 drop of reagent C+20 μl of DAB). After washing with tap water to stop the chromogenic reaction, the sections were dehydrated in an ascending alcohol gradient (10 min per step), cleared twice with xylene (10 min each wash) and mounted in neutral balsam. Then, the sections were examined and imaged.

### Statistical analysis

All experimental data are expressed as the mean±s.e.m. A two-sided Student's *t*-test was applied to estimate the differences between data groups, while multiple group comparisons were conducted by ANOVA. *P*-values <0.05 were considered as statistically significant, and all analyses were performed with SPSS 18.0 software (SPSS Inc., Chicago, IL, USA).

## Figures and Tables

**Table 1:** Primer sequences used for RT–PCR

*Gene*	*Primer sequences*	
	*Forward*	*Reverse*
E-cadherin	5′-CGAGAGCTACACGTTCACGG-3′	5′-GGGTGTCGAGGGAAAAATAGG-3′
Vimentin	5′-GCCCTAGACGAACTGGGTC-3′	5′-GGCTGCAACTGCCTAATGAG-3′
N-cadherin	5′-CAGGGACCAGTTGAAGCACT-3′	5′-TGCCGTGGCCTTAAAGTTAT-3′
GAPDH	5′-TCTTTTGCGTCGCCAGCCGAG-3′	5′-TGACCAGGCGCCCAATACGAC-3′
IL-17	5′-AGAGATATCCCTCTGTGATC-3′	5′-TACCCCAAAGTTATCTCAGG-3′

Abbreviations: GAPDH, glyceraldehyde-3-phosphate dehydrogenase; IL-17, interleukin-17; RT–PCR, reverse transcription PCR.
