# Supplementary figures and images for: The promotion of the transformation of quiescent gastric cancer stem cells by IL-17 and the underlying mechanisms
Source: Oncogene. 2016 Aug 15;36(9):1256–64. doi: 10.1038/onc.2016.291 (PMC5340802; doi:10.1038/onc.2016.291)

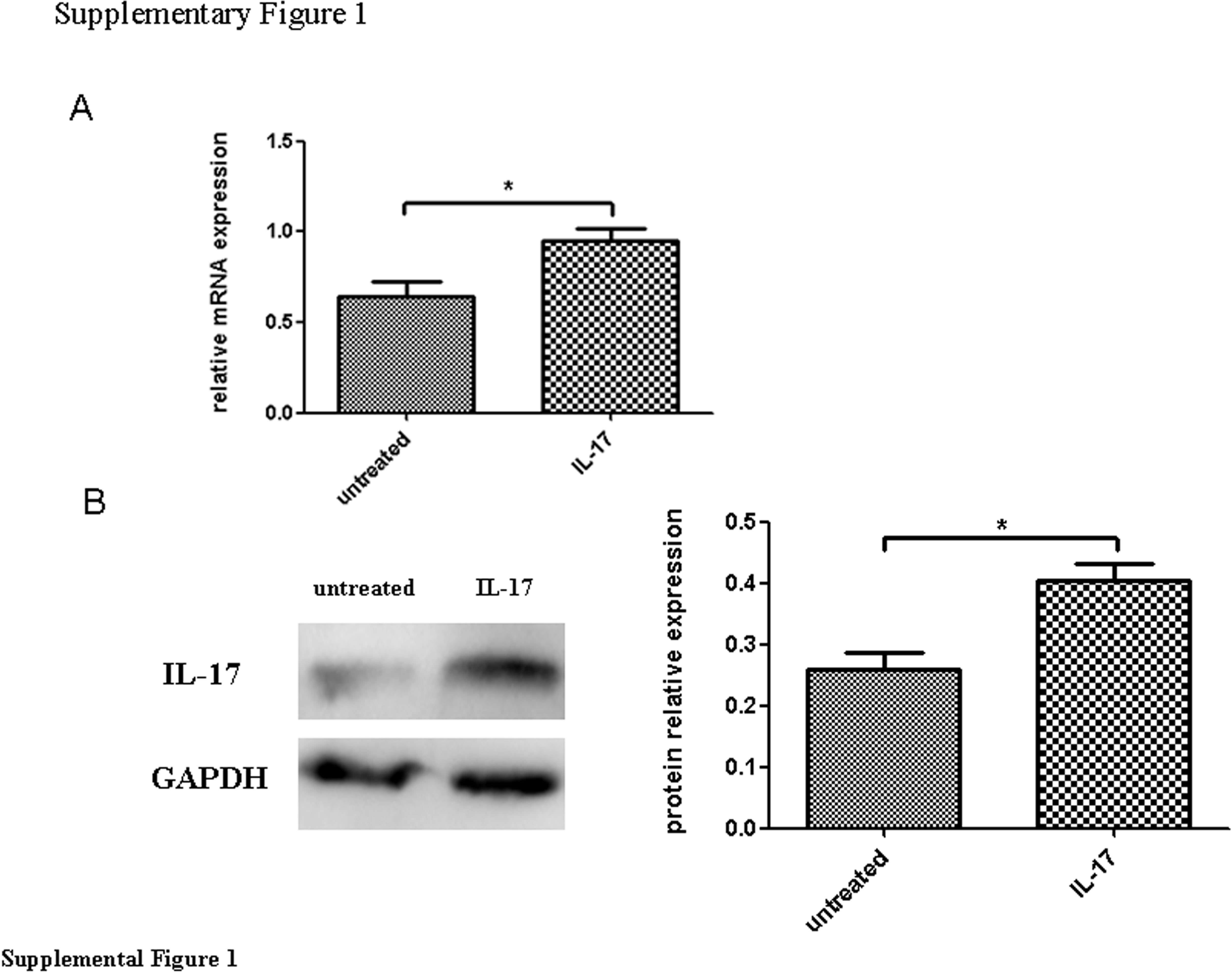

Supplement: Supplementary Figure 1 [file onc2016291x1.tif]

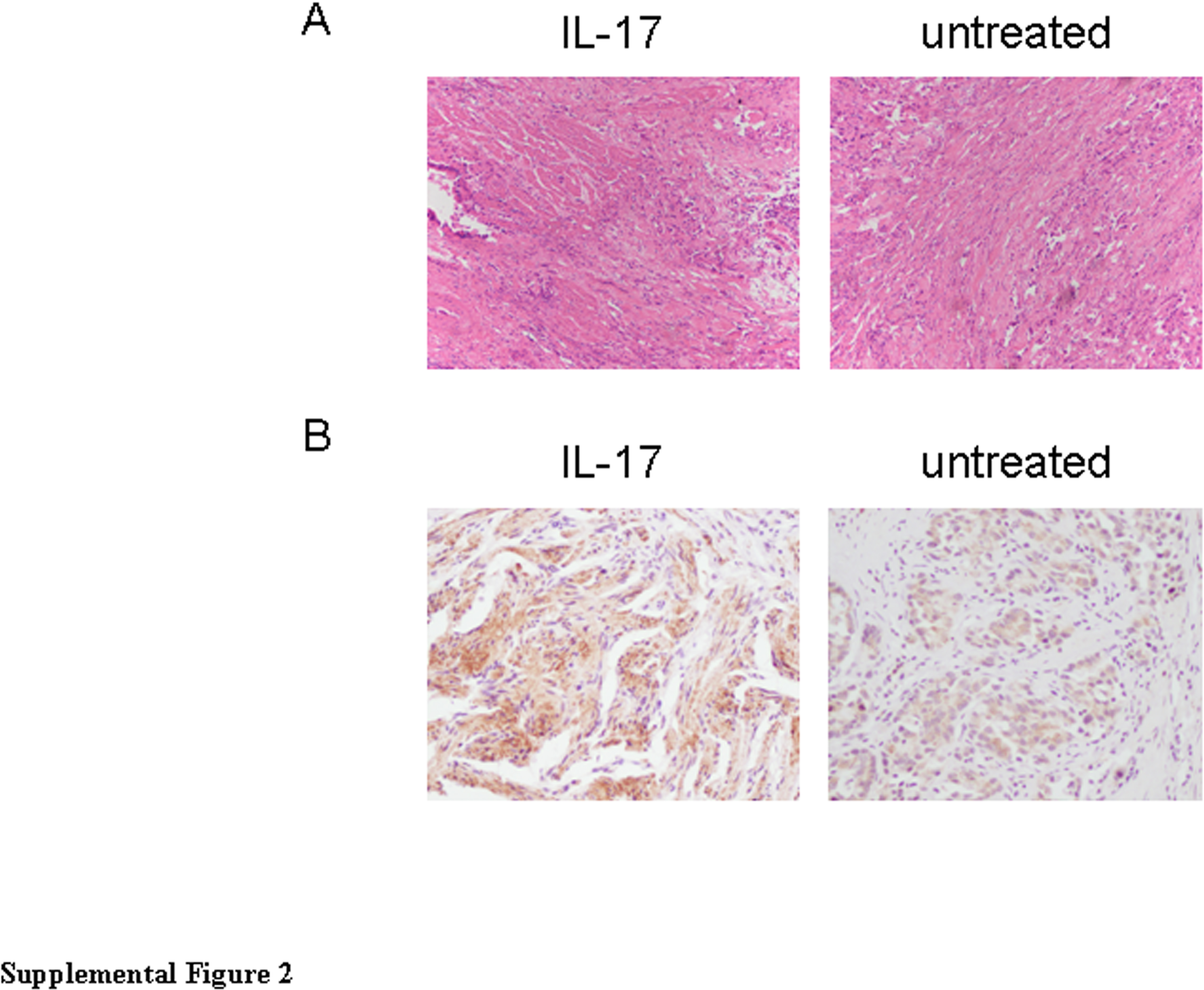

Supplement: Supplementary Figure 2 [file onc2016291x2.tif]
