# Supplementary material for: The promotion of the transformation of quiescent gastric cancer stem cells by IL-17 and the underlying mechanisms
Source: Oncogene. 2016 Aug 15;36(9):1256–64. doi: 10.1038/onc.2016.291 (PMC5340802; doi:10.1038/onc.2016.291)
Supplement: Supplementary Figure Legends [file onc2016291x3.doc]

**Supplementary Figure Legends**

**Fig S1**. Established quiescent gastric cancer stem cells overexpress IL-17. A. IL-17 overexpression was detected by RT-PCR in quiescent gastric cancer stem cells. B. IL-17 overexpression was detected by western blotting analysis in quiescent gastric cancer stem cells.

**Fig S2**. A. H&E staining of the subcutaneous xenograft demonstrated that the tumor were derived from the quiescent GCSCs*.* B. Immunohistochemical staining of the subcutaneous xenograft demonstrated that the tumor were derived from the quiescent GCSCs by IL-17*.*
